# Tracing back ancient oral microbiomes and oral pathogens using dental pulps from ancient teeth

**DOI:** 10.1038/s41522-016-0008-8

**Published:** 2016-12-07

**Authors:** Nicolás Rascovan, Hong Huynh, Gérard Chouin, Kolawole Adekola, Patrice Georges-Zimmermann, Michel Signoli, Yves Desfosses, Gérard Aboudharam, Michel Drancourt, Christelle Desnues

**Affiliations:** 1 Aix Marseille University, URMITE, UM63, CNRS 7278, IRD 198, INSERM, Marseille, France; 2 Lyon G. Tyler Department of History, William & Mary, Virginia, USA; 3 Department of Archaeology and Anthropology, University of Ibadan, Ibadan, Nigeria; 4 INRAP, UMR 5608, TRACES, Toulouse, France; 5 Department of Medicine, Aix Marseille University, UMR 7268 ADES, EFS CNRS, Secteur Nord, Marseille, France

## Abstract

Ancient dental pulps are highly precious samples because they conserve DNA from humans and blood-borne pathogens for ages. However, little is known about the microbial communities present in dental pulps. Here, we analyzed ancient and modern dental pulp samples from different time periods and geographic regions and found that they are colonized by distinct microbial communities, which can be differentiated from other oral cavity samples. We found that despite the presence of environmental bacteria, ancient dental pulps conserve a clear and well-conserved record of oral microbes. We were able to detect several different oral pathogens in ancient and modern dental pulps, which are commonly associated with periodontal diseases. We thus showed that ancient dental pulps are not only valuable sources of DNA from humans and systemic infections, but also an open window for the study of ancient oral microbiomes.

The dental pulp is the internal portion of teeth that contains highly vascularized soft tissues, which are protected by hard and mineralized structures (cementum, enamel, and dentin). Thanks to this protection, dental pulps are exceptional sources of ancient DNA, not only human but also from pathogens that produce systemic infections.^1–4^ In the past few years, several works have used high-throughput sequencing on ancient dental pulp samples to reconstruct the genomes of ancient pathogenic bacteria such as *Yersinia pestis* (the etiological agent of plague, including the Black Death), which dates back to centuries and millennia before the present.^2,5,6^ However, the microbial communities found in living dental pulps have been scarcely studied and it is not known whether ancient dental pulps also keep a record of oral bacteria and periodontal diseases from the past.

Here, we analyzed 16S rRNA amplicon sequencing data sets from ancient and modern dental pulps and provide an unprecedented exploration of their associated microbiomes. We combined these samples with other data sets generated by different laboratories and grouped them in eight categories: (1) Ancient dental pulps, (2) Modern dental pulps, (3) Modern root canals (which are equivalent to dental pulps, but without teeth extraction), (4) Ancient complete teeth that were ground and homogenized, (5) Modern oral cavity surfaces, (6) Saliva, (7) Ancient dental calculus, and (8) Soils (see Table 1 and the methods section from supplementary text for detailed sample description).

**Table 1:** Description of the samples and data sets used in this study

Sample type	Origin	Origin	Period/yearsbefore present	Numberof samples	Reference	PUBMED ID	SRA accession
Completeground teeth	Published works	England	750–650	6	Adler et al.^9^	23416520	ERP002107
		Germany	1000–400				
			7400–6725				
			4150–3600				
		Germany	800–1100	14	Warinner et al.^8^	24562188	SRP029257
Dental root canal		Brazil	Modern	17	Santos et al.^10^	22132218	ERP000669
Dental pulp	This study	France	First World War (WWI)	6	N/A	N/A	SRP068830
		Nigeria	500	15			
		France	Modern	2			
Dental calculus	Published works	England	750–650	55	Adler et al.^9^	23416520	ERP002107
		Germany	1000–400				
			1100–850 4100–2800				
			4150–3600 4450–4000				
			7400–6725 7550–5450				
		Germany	800–1100	4	Warinner et al.^8^	24562188	SRP029257
Saliva		US	Modern	29	Pride et al.^11^	22158393	SRA024393
		US	Modern	9	The HMP (Gevers et al.)^12^	22904687	SRP002395
Oral cavity (diverse regions)		US	Modern	68	The HMP (Gevers et al.)^12^	22904687	SRP002395
Soil		Germany	Modern	18	Will et al.^13^	20729324	SRA020168
		Different environments	Modern	151	Bates et al.^14^	21085198	N/A

A beta-diversity analysis based on the unweighted Unifrac (UU) distance showed an overlap between modern and some of the ancient dental pulp samples, which were markedly different from ancient dental calculus, modern saliva, and oral cavity samples (Fig. 1a). A second group of ancient dental pulp samples clustered together with ancient complete ground teeth and closer to soil than to other samples from the oral cavity. To further explore the differences among microbial communities of the eight groups of samples, we performed a pairwise ANalysis Of SIMilarity (ANOSIM) statistical test^7^ and we obtained low *R*-values (the lower the *R*, the less significant are the differences between communities) when modern root canal samples and modern dental pulps were compared to ancient dental pulp samples (Fig. 1a, upper panel). Similarly, ancient ground teeth also showed low *R*-values when compared to ancient dental pulps, probably indicating a significant contribution of the dental pulp fraction to the microbial profile found in the complete teeth preparation. Overall, these results suggested that dental pulps samples contained distinct microbial communities that differed from other oral cavity samples, and which could be detected even centuries after death.

**Fig. 1:**
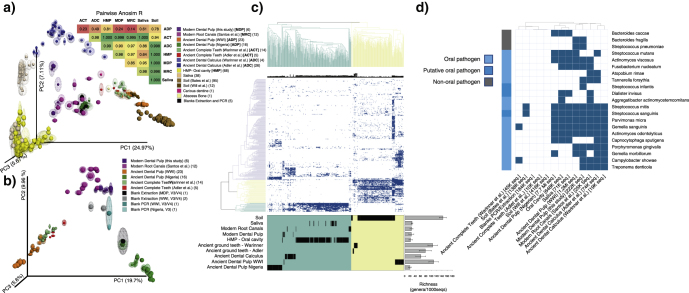
Tracking back oral microbes in ancient and modern dental pulps. **a** PCoA based on UU distances using a reference-based OTU table. Distances between samples were calculated on 100 repetitions of evenly rarefied OTU tables and the halo around each sample indicate the dispersion among repetitions. Samples were colored according to the data set of origin and the references are embedded in the figure. *Bigger spheres* correspond to long amplicons (V3/V4 region of the 16S rRNA) and *smaller spheres* to short amplicons (V3 region). Numbers in *parentheses* indicate the number of samples for each data set and the general category of each is indicated in *square brackets*. Using the UU distance matrices, the eight main categories of samples were mutually compared using a pairwise ANalysis Of SIMilarity (ANOSIM) statistical test and the resulting *R*-values are shown on top of PCoA. The *p*-values obtained in all analyses were always lower than 0.01. **b** All teeth samples were retrieved from the reference-based OTU table, and the resulting table was evenly rarefied 100 times. The UU distance was calculated on these repetitions and plotted using PCoA using same procedures as in **a**. **c** Heatmap analysis showing the genera detected (presence/absence) in at least 10 samples from the total analyzed. All sequences from each data set were used in the analysis. Genera found in blanks of polymerase chain reaction (PCR) and extraction were excluded from the analysis. The Jaccard distance was used to sort samples and genera. Richness was estimated using an even rarefaction of the reference-based OTU table at 1000 sequences per sample and counting the number of known genera observed for each sample. Unknown or uncultured environmental bacteria were not considered. Richness values and error bars correspond to averages and standard deviations, respectively, between all samples within a category. **d** All sequences from each data set were compared by BlastN against the 16S rRNA sequences of known pathogens in PATRIC database. The numbers of sequences used in the analysis are indicated in *square brackets*. Only sequences that showed a 100 % similarity against a pathogen sequence with at least 60 % of the read aligned were considered. As an additional filter, these sequences were then compared against the full NCBI database and only sequences that showed 100 % similarity against non-ambiguous species (i.e., only showed perfect hit against the same species that in PATRIC DB) were conserved. Bacteria that are commonly associated with oral pathologies are indicated in *light blue* on the left of the figure. Three bacterial species that are commonly isolated from the oral cavity but their role in oral pathogenesis has been only suspected are colored in *dark blue*. Pathogens from non-oral pathologies are indicated in *gray*. Samples were grouped by data set and a positive hit was considered when a pathogen was confidently found in at least one sample from that data set

When only dental pulps and complete ground teeth were analyzed, we found three well-defined clusters, one containing modern dental pulp and modern root canal samples, one containing ancient dental pulps and complete ground teeth from European samples of different periods of time (Table 1 for details), and one containing ancient Nigerian dental pulp samples (Fig. 1b). Although the perspective of the principal coordinate analysis (PCoA) in Fig. 1a seemingly shows an overlap between blanks of extraction and polymerase chain reaction (PCR) (the only 5 that amplified from the 12 blanks tested) with some dental pulp samples, Fig. 1b shows that these blanks were clearly different from samples. Our results indicate that although the microbial profiles of ancient and modern dental pulps are close when contrasted to samples of different nature, significant differences can be found between dental pulp samples from different sources. The variations observed between different populations and time periods also point out the relevance of extending these characterizations to individuals from different geographical origins.

To explore in detail the taxonomic composition in the different groups of samples, we analyzed the complete data sets classified at genus level (Fig. 1c). All genera detected in the five blanks mentioned above were excluded from the analysis. We found two main and distinct clusters, one containing soil samples (in yellow) and one containing samples retrieved from the oral cavity (saliva and oral sites, in green), with several genera that were mutually exclusive between both groups. In agreement with results from Fig. 1a, ancient dental pulps and complete ground teeth from European samples showed a higher degree of environmental contamination, with several genera that are typically found in soil but not in the oral cavity and modern dental pulps, as well as intermediate richness values between both groups. However, despite the presence of these contaminants, we could detect a clear signal of genera that are typically found in oral samples and are totally absent in soil. On the other hand, ancient dental pulps from Nigeria, which also showed a clear record of oral bacteria, showed a much lower sign of contamination and also presented several genera that were exclusively found in these samples. Since ancient African teeth have never been analyzed before, we cannot rule out the possibility that the latter is due to particularities in the oral microbiome of these populations. Using a Kruskal–Wallis statistical test, we could first identify which genera presented significant differences between modern oral microbiomes and soils and then estimate the proportion of genera in ancient samples (dental pulps, complete ground teeth, and dental calculus) presenting oral-like profiles (see Supplementary results and Supplementary Table 1 in the Supplementary information file for further detail). We found that 30–50 % of the oral-associated genera were also enriched in ancient dental pulp, a proportion that was much higher than in complete ground teeth and relatively similar to that found in dental calculus (Supplementary Table 1). The statistical analyses also revealed a number of genera that were not associated with modern oral samples, but that showed significantly higher proportion in ancient samples compared to soil. These genera could potentially correspond to either bacteria-enriched post-mortem that may participate in decomposition or, alternatively, particular residents of the ancient oral microbiomes analyzed in this study (Supplementary Tables 2 and 3). All together, our results indicate that although ancient dental pulps are invaded by environmental bacteria after death, they also conserve a good record of the microbial communities that are naturally present in the oral cavity and particularly in dental pulps.

We finally explored the presence of pathogenic bacteria in all groups of samples (i.e., unambiguous hits against known pathogens). We found almost exclusively hits against oral pathogenic bacteria (Fig. 1d) from 18 different species, a group that is not overrepresented in the database used. From these, 12 were found in at least one ancient sample. The analysis of individual samples showed a high variability, with positive hits in only a limited number of ancient samples (Supplementary Figure 1). This is probably determined by the natural variability in the prevalence of oral pathogens among individuals and even among teeth of the same oral cavity, although it could also be influenced by the conservation of bacterial DNA in these samples. We did not detect any systemic pathogen that could have likely caused the death of any of the analyzed individuals. We therefore demonstrate that ancient dental pulps can also conserve a good record of ancient oral pathogens that were likely producing localized bacteremia. These results are in agreement with previous studies showing that modern oral pathogens were already present in ancient populations.^8,9^ Here, we showed the first evidence that DNA from oral pathogens can be also recovered from ancient dental pulp samples.

In this work we showed that dental pulps are not only highly precious sources of ancient human and blood-borne pathogens DNA, but also from the oral microbes that prevailed in association with our ancestors. This work brings new perspectives for studying the evolution of human oral microbiomes and the spread of periodontal diseases in ancient populations, through dental pulp metagenomics.

## Electronic supplementary material

Supplementary Information
