# Supplementary material for: Tracing back ancient oral microbiomes and oral pathogens using dental pulps from ancient teeth
Source: NPJ Biofilms Microbiomes. 2016 Dec 7;2:6. doi: 10.1038/s41522-016-0008-8 (PMC5460193; doi:10.1038/s41522-016-0008-8)
Supplement: Supplementary file 1 — Supplementary Information [file 41522_2016_8_MOESM1_ESM.docx]

**Tracing back ancient oral microbiomes and oral pathogens using dental pulps from ancient teeth**

Nicolás Rascovan, Hong Huynh, Gérard Chouin, Kolawole Adekola, Patrice Georges-Zimmermann, Michel Signoli, Yves Desfosses, Gérard Aboudharam, Michel Drancourt, Christelle Desnues.

**Supporting Information:**

**Supplementary Information Content:**

Supplementary Materials and Methods

Supplementary Results

Supplementary References

Supplementary Figure S1

Supplementary Table S1

Supplementary Table S2

Supplementary Table S3

**Supplementary Materials and Methods:**

**Sample description**

*Dental pulps from Nigeria:*

We processed 15 teeth obtained from different individuals found in a mass grave excavated in 1962/1963 by Graham Connah at Benin City, on the Clerk’s Quarters site ^1^. The remains were found in the fill of a well-like cistern recorded as “feature 21”, in Cutting II. On the basis of the number of femora present, it was established that the mass grave contained skeletal remains of at least 41 individuals. Although exceptionally well preserved, the skeletal material was treated “by immersion in some cases in a solution of Bedacryl and Toluol, and in others in a solution of Durofix and acetone” (Connah 1975: 62 ^1^). Examination of the remains suggested that all but one of the individuals were female and between 15 and 35 years old. Radiological examination suggested that this range could be narrowed to perhaps 19-24 years. The bones gave no clue as to the cause of death. Two charcoal samples recovered amidst the bones in feature 21 provided two radiocarbon dates suggesting a 13th–14th century date for the remains. After analysis, the human remains were kept in wooden boxes at the Department of Anatomy and then transported to the storage unit of the Department of Archaeology at the University of Ibadan, where the authors re-discovered them in 2013. Dental material was taken and exported for processing with the authorization of the head of the Department of Archaeology and Anthropology at the University of Ibadan. Whereas Connah suggested that the formation of such a mass grave could have been the result of human sacrifices, we think that it cannot be ruled out the possibility of an epidemic to explain the death of these individuals. We believe that both the absence of any violent injury and the fact that the bodies were associated with textiles and bronze jewelry, typically associated with elite groups, are possible evidences of a catastrophic event, such as an epidemic, which may have struck a group of segregated royal wives within the palace complex ^2^. However, in the absence of direct evidences found neither in previous works nor in the present study, we cannot be conclusive on any cause of death.

*Dental pulps from World War I:*

In 2012, during an archaeological excavation operation in the commune of Saint-Leonard (outside Reims), several series of "foxholes" from the beginning of the First World War were uncovered. Some of these individual holes contained the bodies of German soldiers. Together with the human remains, a piece of helmet and a belt belonging to the 3rd Regiment of foot from the Imperial German Guard quickly helped guide the historical investigation. This regiment was engaged in a furious fighting at the bridge of Saint-Léonard, spanning at the Marne canal in Aisne in September 26, 1914. This attack, blocked by French troops, caused significant losses in the German regiment and several bodies were left on the field during a retreat at night ^3^. Two independent teeth from three different skulls were recovered from these remains and used for analyses of the dental pulps.

*External datasets:*

External datasets were obtained from previously published and publicly available data ^4-11^. We used a total of 373 samples for the analyses: 59 from ancient dental calculus (55 from Adler et al. ^8^ and 4 from Warinner et al. ^7^), 20 from ancient teeth (6 from Adler et al. ^8^ and 14 from Warinner et al. ^7^), 17 from modern teeth (Santos et al. ^4^), 38 from saliva (29 from Pride et al. ^10^ and 9 from the Human microbiome project ^11^), 169 from soil (151 from Bates et al. ^5^ and 18 from Will et al. ^6^), 1 carious dentine and 1 abscess bone from Warinnet et al. ^7^, and 68 from the oral cavity obtained from the Human microbiome Project (attached keratinized gingiva 10, buccal mucosa 10, hard palate 10, palatine tonsils 10, supragingival plaque 10, throat 8, tongue dorsum 10, equally distributed between males and females) ^11^.

The number of samples from each dataset and corresponding dating are detailed in Table 1.

**Sample processing and sequencing**

After selection of teeth (with no decay, or traumatic injury and a closed apex), the outer surface of each tooth was thoroughly cleaned with sodium hypochlorite and was then fractured longitudinally along the long axis. The dental pulp powdery residues were scraped from the pulp cavities with an excavator and placed in sterile tubes and DNA was prepared using a phenol/chloroform extraction. All manipulations of ancient teeth, including opening and collection of the pulps, were performed with sterile reagents and carefully cleaned material in a different building to avoid cross contamination.

Modern teeth were initially extracted from patients for strategic reasons (prosthetic treatment according to patient's interest) or for periodontal reasons (mobility). After extraction, teeth were placed in sterile Phosphate-buffered saline buffer (PBS) and DNA was extracted as soon as possible following the same procedures used for ancient dental pulps. All patients gave their consent for using these teeth for research purposes.

DNA amplification was performed in a laboratory with biosafety level 3, which was specially prepared for ancient DNA work. All surfaces were cleaned with DNAzap reagent (Invitrogen AM9890), all reagents and disposable material were new and a dedicated set of new pipettes was used exclusively for non-amplified ancient DNA work. Amplification was performed using the following mix: Buffer HF 5X: 4 µl, BSA 2.5 mg/ml: 1ul, dNTPs 10mM: 0.4 µl, Water: 10.4 µl, Primer mix (10uM each): 2 µl, DNA solution: 2 µl and Phusion Hot Start II: 0.2 µl (Thermo Scientific F549L). We used two sets of primers, one covering the V3 region of the 16S rRNA gene (short amplicons) from Adler et al. ^8^ and one covering the V3/V4 region (long amplicons) from Klindworth et al. ^12^. Both set of primers were fused to Illumina adapters following manufacturer indications. The two amplification programs were, A) V3-V4 long F341-R785: 98˚C 30 sec, 45 x (98˚C 10 sec, 64˚C 30 sec, 72˚C 20 sec), 72˚C 10 min and B) V3-short F351-R507: 98˚C 30 sec, 45 x (98˚C 15sec, 65˚C 20 sec, 72˚C 20 sec), 72˚C 5 min. All PCR reactions were performed in triplicate to minimize biases due to the PCR amplification. Additionally, to discard biases due to the number of amplification cycles, few samples were amplified using a lower number of cycles (32 cycles). In all procedures we used blanks of extraction (5 samples) and a blanks of PCR (7 samples). From these 12 blanks, amplification occurred in only five tubes, with a significantly lower yield than the obtained with dental pulp samples. The amplified and sequenced blanks were: Blank PCR Nigeria samples V3 primers, Blank Extraction WWI samples V3/V4 primers at 32 cycles and 45 cycles of PCR, Blank PCR WWI samples V3/V4 primers, Blank Extraction Modern samples V3/V4 primers. Library preparations were finished following Illumina protocols and sequenced in an Illumina MiSeq instrument using 2x250bp pair-end sequencing. Raw data has been deposited in the SRA archive under the accession number SRP068830.

**Data processing**

Raw data was processed and analyzed using QIIME v1.9 software ^13^. R1 and R2 reads of long V3/V4 amplicons were assembled using the join_paired_ends.py script (although they were analyzed separately for certain analyses). Since short V3 amplicons were shorter that the single pair read, the R1 and R2 reads were treated separately in this case. Primer trimming and quality filtering was performed with the split_libraries_fastq.py script with a limit of 2 maximum consecutive low quality bases, 90% of high quality base calls and a minimum quality value of 29. Long assembled amplicons shorter than 400 bp and short amplicons shorter that 120 bp were discarded. External datasets were trimmed using the same parameters.

**Data analysis**

For beta-diversity analysis, amplicon datasets covering the V3 and the V3/V4 regions were initially processed separately using a *de novo* OTU picking method. We standardized large datasets to 3,000 sequences per sample. Sequences were clustered using the uclust method with optimal parameters. We then combined short and long amplicon data using a reference-based OTU picking method. To do so, we performed a BlastN analysis of the *de novo* OTU representative sequences from short and long amplicon datasets against the Silva Database (release 119) clustered at 97% similarity, and then combined *de novo* OTU tables and collapsed them based on common best hits in Silva DB, a similar strategy to that used in previous works ^14^. Hits with less than 97% similarity were discarded. Reference-based OTU representative sequences (the Silva DB sequences) were aligned using the PyNast method. Phylogenetic trees from OTU representative sequences were constructed using the fastree method. The OTU table was rarified at 800 sequences per sample (the value was constrained by the limited number of sequences in some external datasets) with 100 repetitions to avoid biases in the subsampling. The distance between samples was estimated using the unweighted Unifrac metric, which takes into account not only the collection of observed OTUs, but also the phylogenetic structure of the community. Since stochastic degradation processes are likely to affect relative abundances of microbial taxa in ancient samples, we only determined taxa composition at a qualitative level (presence/absence). We confirmed the validity of the reference-based OTU picking method by comparing the obtained distance matrices with those obtained by *de novo* OTU picking (for V3 and V3/V4 amplicon datasets, separately), an analysis that did not show significant differences (Mantel test R>0.9, p=0.01).

For ANOSIM pairwise analysis we used a strategy previously used in the literature ^15^. The 439 samples were grouped according to five categories (ancient teeth, modern teeth, ancient dental calculus, HMP-oral, saliva and soil). Then the ANOSIM statistical test was calculated on each pair of groups to test whether samples from both groups were significantly different. We performed this test on 100 repetitions of rarefied OTU tables (with 800 sequences per sample) and calculated the average value of R (repetitions showed almost identical R values).

To build the heatmap presented in Figure 1C, we classified all sequences available from each dataset using the RDP classifier on the Greengenes database included in QIIME v1.9. Samples with less than 800 sequences were excluded from the analysis. We grouped sequences according to genus level classification (only those classified at specific genera were conserved) and then conserved only genera that were found in at least 10 samples. All genera detected in blanks of extraction and PCR were excluded from the analysis. The resulting table was transformed into presence/absence values and heatmaps were constructed using the annHeatmap2 function in R. Distances and dendrograms were calculated using the Jaccard distance (recommended for presence/absence tables) included in the Vegan package.

We used the Kruskal-Wallis non-parametric test included in QIIME software on the filtered table from Figure 1C (with relative abundances) to identify genera that presented significant differences (Bonferroni corrected p-value < 0.05) between modern oral samples (dental pulp, saliva and other oral cavity areas) and soil (considered here as source of environmental contamination). We separated the resulting genera in three groups: those enriched or exclusively present in 1) Modern oral cavity (Supplementary table 1); 2) Soil (Supplementary table 2); 3) with no significant differences between both groups (Supplementary table 3). We then used the same test to compare each group of ancient samples (Dental pulps from Nigeria samples, Dental pulps from WWI samples, Ground teeth from Adler et al., Ground teeth from Warinner et al. and Dental Calculus from Adler/Warinner combined) to soil samples to identify genera that may have remained from oral microbiomes and those that likely correspond to environmental contaminations. Supplementary tables show those genera that presented significant differences in at least one comparison against soil samples and values correspond to significant Bonferroni corrected p-values (< 0.05).

To evaluate genera richness, the reference-based OTU table was collapsed according to genera classification and all fields including uncultured and unclassified sequences were discarded (only known genera were kept). The resulting table was rarefied at 1000 sequences per sample and the number of observed genera was quantified on each sample. The average values and corresponding standard deviation was calculated for each group of samples.

To search for known pathogens, we downloaded the 16S rRNA data from the PATRIC database ^16^ and selected only those that are annotated as human pathogens. Since we were interested in detecting the presence of bacterial taxa that are expected to be in low abundance, all sequences from each dataset were used for this analysis (totaling 12,134,174 reads). We then performed a BlastN analysis of all sequences against the pathogen database. For long amplicons, R1 and R2 were blasted separately because mis-assemblies could have affected results. We selected only reads with 100% similarity to best hit with alignments covering more than 60% of the read. To validate hits, we recovered the aligned part of these reads and blasted them against the NCBI database. We retrieved NCBI hits with 100% similarity in the total length of the sequence (i.e., identical alignment length and % similarity as in the best hit obtained against PATRIC database) and collected all the TaxID obtained. Finally we only conserved those sequences that returned a single TaxID (or several redundant annotations) with the same annotation as in the PATRIC hit. Reads meeting these conditions were considered unequivocal pathogens hits.

**Supplementary Results:**

A total of 10,329 OTUs were found by the reference-based OTU picking method. These were collapsed into 2,408 groups based on the taxonomic classification (up to genus level), although many of them corresponded to consensuses at higher taxonomic ranks than genus (e.g., up to family, order, class, etc.) or to unclassified taxa. This number was reduced to 792 when samples with less than 800 sequences were eliminated as well as those consensus groups with classifications that did not correspond to precise genera. Finally, after eliminating those genera that were observed in less than 10 samples (from the 547 remaining at this point) and those genera that were observed in blanks of PCR and extraction, we conserved a total of 300 genera. Figure 1C was constructed using a presence/absence version of this table.

We then used a Kruskal-Wallis statistical test to identify the genera that presented significant differences between modern oral cavity samples (i.e., dental pulps, saliva and other oral cavity surfaces) and soils (which are considered as the main source of environmental contamination). We found that a total of 65 genera presented a significantly higher abundance in at least one group of oral cavity samples (Supplementary Table 1), whereas 128 were more abundant in soil (Supplementary Table 2). We then used the same test to determine which of these genera also presented significant differences between soil and each particular group of ancient samples (i.e., Dental pulps from Nigeria samples, Dental pulps from WWI samples, Ground teeth from Adler et al., Ground teeth from Warinner et al. and Dental Calculus from Adler/Warinner combined).

Our results showed that among the 65 oral-associated genera, 32 and 22 were also significantly higher in ancient dental pulps from WWI and Nigeria respectively (Supplementary table 1). On the other hand, each group of ancient ground teeth (from Warinner and Adler studies) showed only 4 out of 65 genera with significantly higher abundance than in soil samples, while ancient dental calculus showed the highest number of oral-associated genera (40). These results strongly support our claim that dental pulps conserve a good record of oral microbes. They also suggest that this record is significantly superior that the recovered from grinding complete teeth and close to that recovered from ancient dental calculus.

As an additional support, when the 128 soil-associated genera were analyzed (i.e., genera with significantly higher abundance in soil than in modern oral cavity), we found that nearly 40% of these genera also showed a significantly lower abundance in ancient dental pulps than in soil (Supplementary table 2). However, for the case of complete ground teeth only 9% of the 128 genera showed a significantly lower abundance than in soil for Warinner et al. samples and none for Adler et al. samples. These results could indicate that either ancient ground teeth contain a much higher predominance of soil bacteria or a significantly poorer representation of oral bacteria.

Interestingly, several genera found in ancient samples (dental pulps, ground teeth and calculus), showed not only significant differences with soil samples, but also opposite results compared to modern oral samples (Supplementary table 2, cells marked in green). These cases correspond to genera that were significantly enriched in ancient samples compared to soil, while they normally present a significantly lower abundance in modern oral samples. Moreover, among the genera that did not show significant results between soil and modern oral samples, there were 58 genera that were more abundant in at least one of the groups of ancient samples (Supplementary table 3). Taking these results into account, we can hypothesize that these genera might correspond to bacteria that got enriched post-mortem, which may also be involved in the process of decomposition. Nevertheless, we cannot rule out the possibility that these genera correspond to particular residents of the ancient oral microbiomes of the individuals analyzed in this study.

**Supplementary References:**

1 Connah, G. *The Archaeology of Benin. Excavations and other researches in and around Benin City, Nigeria*. (Clarendon Press (Oxford University Press), 1975).

2 Chouin, G. L. F. *Fossés, enceintes et peste noire en Afrique de l’Ouest forestière (500-1500 AD)*. Vol. 9 43-66 (CNRS Editions, 2013).

3 Desfossés, Y. & Signoli, M. in *La Grande Guerre des Corps : Des Corps en Guerre* Vol. 12 *Corps* 15-25 (CNRS Editions, 2015).

4 Santos, A. L. *et al.* Comparing the bacterial diversity of acute and chronic dental root canal infections. *PloS one* **6**, e28088, doi:10.1371/journal.pone.0028088 (2011).

5 Bates, S. T. *et al.* Examining the global distribution of dominant archaeal populations in soil. *The ISME journal* **5**, 908-917, doi:10.1038/ismej.2010.171 (2011).

6 Will, C. *et al.* Horizon-specific bacterial community composition of German grassland soils, as revealed by pyrosequencing-based analysis of 16S rRNA genes. *Applied and environmental microbiology* **76**, 6751-6759, doi:10.1128/AEM.01063-10 (2010).

7 Warinner, C. *et al.* Pathogens and host immunity in the ancient human oral cavity. *Nature genetics* **46**, 336-344, doi:10.1038/ng.2906 (2014).

8 Adler, C. J. *et al.* Sequencing ancient calcified dental plaque shows changes in oral microbiota with dietary shifts of the Neolithic and Industrial revolutions. *Nature genetics* **45**, 450-455, 455e451, doi:10.1038/ng.2536 (2013).

9 Human Microbiome Project, C. Structure, function and diversity of the healthy human microbiome. *Nature* **486**, 207-214, doi:10.1038/nature11234 (2012).

10 Pride, D. T. *et al.* Analysis of streptococcal CRISPRs from human saliva reveals substantial sequence diversity within and between subjects over time. *Genome research* **21**, 126-136, doi:10.1101/gr.111732.110 (2011).

11 Gevers, D. *et al.* The Human Microbiome Project: a community resource for the healthy human microbiome. *PLoS biology* **10**, e1001377, doi:10.1371/journal.pbio.1001377 (2012).

12 Klindworth, A. *et al.* Evaluation of general 16S ribosomal RNA gene PCR primers for classical and next-generation sequencing-based diversity studies. *Nucleic acids research* **41**, e1, doi:10.1093/nar/gks808 (2013).

13 Caporaso, J. G. *et al.* QIIME allows analysis of high-throughput community sequencing data. *Nature methods* **7**, 335-336, doi:10.1038/nmeth.f.303 (2010).

14 Caporaso, J. G. *et al.* Moving pictures of the human microbiome. *Genome biology* **12**, R50, doi:10.1186/gb-2011-12-5-r50 (2011).

15 Delsuc, F. *et al.* Convergence of gut microbiomes in myrmecophagous mammals. *Molecular ecology* **23**, 1301-1317, doi:10.1111/mec.12501 (2014).

16 Wattam, A. R. *et al.* PATRIC, the bacterial bioinformatics database and analysis resource. *Nucleic acids research* **42**, D581-591, doi:10.1093/nar/gkt1099 (2014).

**Supplementary Figure S1: Human pathogens detection in ancient and modern teeth, by sample.** The same results as shown for teeth samples in Figure 1D of the paper, but represented by each individual sample instead of grouping by dataset. The intention of the figure is to show the variability among samples from each dataset.

**Supplementary Table S1:** **Genera with significantly higher abundance in modern oral cavity samples than in soil.** The Kruskal-Wallis test was used for the analysis and results were considered as significant when the p-value was lower than 0.05. The statistical test was calculated for each group of ancient and modern samples (columns) against soil and the resulting Bonferroni-corrected significant p-values (p-value < 0.05) are shown. Cells colored in green indicate that the corresponding genera were significantly overrepresented in the corresponding group of samples compared to soil. The total number of genera that presented significant differences for each group of samples is indicated in the top of the table.

**Supplementary table S2:** **Genera with significantly higher abundance in soil than in modern oral cavity samples.** The Kruskal-Wallis test was used for the analysis and results were considered as significant when the p-value was lower than 0.05. The statistical test was calculated for each group of ancient and modern samples (columns) against soil and the resulting Bonferroni-corrected significant p-values (p-value < 0.05) are shown. Cells colored in red indicate that the corresponding genera were significantly overrepresented in soil compared to the corresponding group of samples. Cells colored in green indicate a significantly higher abundance in the corresponding ancient samples compared to soil. The total number of genera that presented significant differences for each group of samples is indicated in the top of the table.

**Supplementary table S3: Genera that did not present significant differences between modern oral cavity and soil samples.** The Kruskal-Wallis test was used for the analysis and results were considered as not significant when the p-value was higher than 0.05. The statistical test was repeated for each group of ancient samples (columns) against soil and the resulting Bonferroni-corrected significant p-values (p-value < 0.05) are shown. Cells colored in green indicate that the corresponding genera were significantly overrepresented in the corresponding group of samples compared to soil. The total number of genera that presented significant differences for each group of ancient samples is indicated in the top of the table.
